# Editorial: Alfred Pingoud (1945–2015)

**DOI:** 10.1093/nar/gkv846

**Published:** 2015-09-18

Scientific colleagues, personal friends and the editors of Nucleic Acids Research were all saddened to learn of the recent death of Dr Alfred M. Pingoud, a long-time contributor to the journal both as a prolific contributor in many areas of nucleic acid enzymology and as a frequent reviewer for the journal.

Dr Pingoud was born on 31 August 1945, as the son of a businessman and a teacher in Schwaan (Mecklenburg, Germany). After completing high school in Langen (Hessen, Germany), he studied Biochemistry at the University of Tübingen and at Amherst College in Massachusetts, graduating in 1969 with a thesis describing ‘Studies on the aldehydeoxidase’ with Prof Ernst Bayer at the Institute of Organic Chemistry.

Alfred was engaged in the study of structure-function relationships of enzymes that interact with nucleic acids, protein design and protein engineering for over 40 years. He began his scientific work with the study of tRNAs and aminoacyl-tRNA synthetases with Prof Günther Maaß in the Department of Biophysical Chemistry at the Society for Biomolecular Research in Braunschweig (Germany) where he received his doctorate in 1973. He then worked at the Hannover Medical School where he finished his habilitation on studies of the function of G-proteins in protein biosynthesis and initiated his own research program. In 1992, he accepted an appointment at the University of Giessen, where he built up the Institute of Biochemistry in the Department of Biology and Chemistry and was its Managing Director until his retirement in 2013. Over the last 30 years of his research, he devoted himself primarily to the study of nucleases from prokaryotes and eukaryotes, starting first with restriction endonucleases, and then later non-specific nucleases and homing endonucleases. He made several highly respected contributions to the enzymology of these enzymes and more recently to programmable nucleases for genome modification. Dr Pingoud published about 300 publications in international scientific journals including 29 in Nucleic Acids Research, the first of which appeared in 1977 (see a full list of his publications in NAR below). Many of those publications were co-authored with his wife, Vera Pingoud, who preceded her husband in death and was also a remarkable biochemical researcher and long-time contributor at NAR.

Dr Pingoud is remembered by his colleagues as an outstanding scientist and human being. He had great analytical skills and a critical mind coupled with an irrepressible curiosity and universal enthusiasm for his science. He drew the people around him under his spell, motivated by his energy and his enthusiasm, his warm-hearted nature, his curiosity and his delight about large and small discoveries. His critiques of his colleagues and their work were always scientifically rigorous and challenging, while also full of respect, empathy and affection. Alfred applied the highest ethical standards to himself and others, and was an admirable role model for generations of students to whom he presented the best side of the scientific world. He was a great communicator who devoted himself to international networking in science as a coordinator of various international programs. People and other cultures he met with the same curiosity, openness and enthusiasm as scientific issues. He not only impressed through his detailed biochemical knowledge, but also via his immense general education and an innate sense of how to express ideas. He intuitively asked the right questions at the right time and had the skills to answer these questions with his work. He took up new developments with enthusiasm, pursuing innovative approaches and with his optimistic perspective he often identified the good results where others despaired. Alfred Pingoud died much too early, only three years after his retirement. The world is a poorer place without him: our deep sympathy and respect is extended to his family.

Albert Jeltsch^1^, Wolfgang Wende^2^, Peter Friedhoff^2^ and Barry L. Stoddard^3^

^1^Institut für Biochemie, Universität Stuttgart, Germany; ^2^Institut für Biochemie JLU Giessen, Germany; and ^3^Fred Hutchinson Cancer Research Center, Seattle WA, USA.


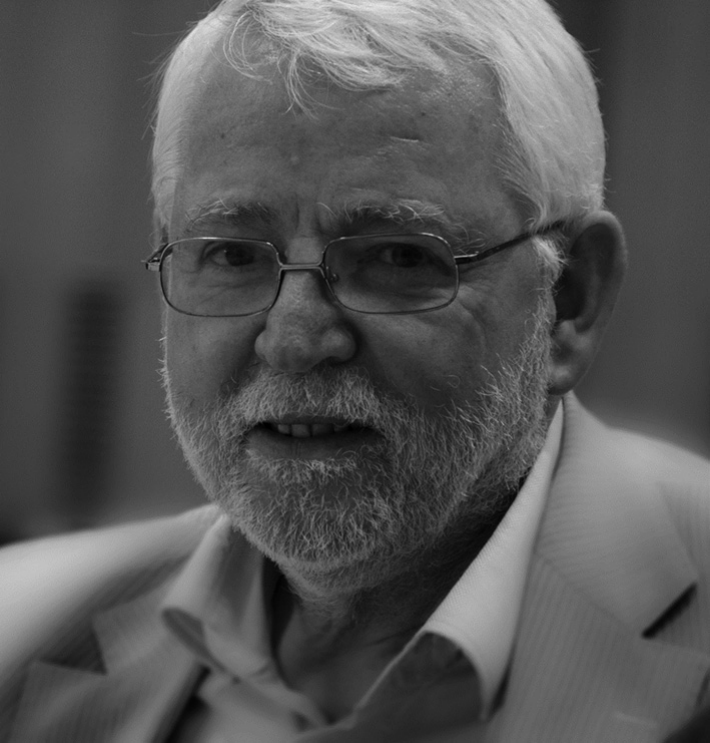


Alfred at the 2010 NEB conference on DNA restriction and modification (Bremen, Germany)

**Publications by Alfred Pingoud and colleagues in *Nucleic Acids Research* (1977–2014)**

1. **Pingoud A**, Kownatzki R, Maass G. (1997) Fluoresceinylthiocarbamyl-tRNATyr: a useful derivative of tRNATyr (*E. coli*) for physicochemical studies. *Nucleic Acids Res.*, **4** (2), 327–338.
2. Langowski J, **Pingoud A**, Goppelt M, Maass G. (1980) Inhibition of Eco RI action by polynucleotides. A characterization of the non-specific binding of the enzyme to DNA. *Nucleic Acids Res*., **8** (20), 4727–4736.
3. Langowski J, Urbanke C, **Pingoud A**, Maass G. (1981) Transient cleavage kinetics of the Eco RI restriction endonuclease measured in a pulsed quench-flow apparatus: enzyme concentration-dependent activity change. *Nucleic Acids Res.*, **9** (14), 3483–3490.
4. Goppelt M, Langowski J, **Pingoud A**, Haupt W, Urbanke C, Mayer H, Maass G. (1981) The effect of several nucleic acid binding drugs on the cleavage of d(GGAATTCC) and pBR 322 by the Eco RI restriction endonuclease. *Nucleic Acids Res.*, **9** (22), 6115–6127.
5. Langowski J, Alves J, **Pingoud A**, Maass G. (1983) Does the specific recognition of DNA by the restriction endonuclease EcoRI involve a linear diffusion step? Investigation of the processivity of the EcoRI endonuclease. *Nucleic Acids Res.*, **11** (2), 501–513.
6. Fliess A, Wolfes H, Rosenthal A, Schwellnus K, Blöcker H, Frank R, **Pingoud A**. (1986) Role of thymidine residues in DNA recognition by the EcoRI and EcoRV restriction endonucleases. *Nucleic Acids Res.*, **14** (8), 3463–3474.
7. Wolfes H, Alves J, Fliess A, Geiger R, **Pingoud A**. (1986) Site directed mutagenesis experiments suggest that Glu 111, Glu 144 and Arg 145 are essential for endonucleolytic activity of EcoRI. *Nucleic Acids Res*., **14** (22), 9063–9080.
8. Fliess A, Wolfes H, Seela F, **Pingoud A**. (1988) Analysis of the recognition mechanism involved in the EcoRV catalyzed cleavage of DNA using modified oligodeoxynucleotides. *Nucleic Acids Res.*, **16** (24), 11781–11793.
9. Friedhoff P, Gimadutdinow O, **Pingoud A**. (1994) Identification of catalytically relevant amino acids of the extracellular Serratia marcescens endonuclease by alignment-guided mutagenesis. *Nucleic Acids Res.*, **22** (16), 3280–3287.
10. Friedhoff P, Kolmes B, Gimadutdinow O, Wende W, Krause KL, **Pingoud A**. (1996) Analysis of the mechanism of the Serratia nuclease using site-directed mutagenesis. *Nucleic Acids Res.*, **24** (14), 2632–2639.
11. Wende W, Grindl W, Christ F, **Pingoud A**, Pingoud V. (1996) Binding, bending and cleavage of DNA substrates by the homing endonuclease Pl-SceI. *Nucleic Acids Res.*, **24** (21), 4123–4132.
12. Grindl W, Wende W, Pingoud V, **Pingoud A**. (1998) The protein splicing domain of the homing endonuclease PI-sceI is responsible for specific DNA binding. *Nucleic Acids Res.*, **26** (8), 1857–1862.
13. Kubareva EA, Thole H, Karyagina AS, Oretskaya TS, **Pingoud A**, Pingoud V. (2000) Identification of a base-specific contact between the restriction endonuclease SsoII and its recognition sequence by photocross-linking. *Nucleic Acids Res.*, **28** (5), 1085–1091.
14. **Pingoud A**, Jeltsch A. (2001) Structure and function of type II restriction endonucleases. *Nucleic Acids Res.*, **29** (18), 3705–3727.
15. Meiss G, Scholz SR, Korn C, Gimadutdinow O, **Pingoud A**. (2001) Identification of functionally relevant histidine residues in the apoptotic nuclease CAD. *Nucleic Acids Res.*, **29** (19), 3901–3909.
16. Korn C, Scholz SR, Gimadutdinow O, **Pingoud A**, Meiss G. (2002) Involvement of conserved histidine, lysine and tyrosine residues in the mechanism of DNA cleavage by the caspase-3 activated DNase CAD. *Nucleic Acids Res.*, **30** (6), 1325–1332.
17. Scholz SR, Korn C, Gimadutdinow O, Knoblauch M, **Pingoud** A, Meiss G. (2002) The effect of ICAD-S on the formation and intracellular distribution of a nucleolytically active caspase-activated DNase. *Nucleic Acids Res.*, **30** (14), 3045–3051.
18. Werner E, Wende W, **Pingoud A**, Heinemann U. (2002) High resolution crystal structure of domain I of the Saccharomyces cerevisiae homing endonuclease PI-SceI. *Nucleic Acids Res.*, **30** (18), 3962–3971.
19. Roberts RJ, Belfort M, Bestor T, Bhagwat AS, Bickle TA, Bitinaite J, Blumenthal RM, Degtyarev SKh, Dryden DT, Dybvig K, Firman K, Gromova ES, Gumport RI, Halford SE, Hattman S, Heitman J, Hornby DP, Janulaitis A, Jeltsch A, Josephsen J, Kiss A, Klaenhammer TR, Kobayashi I, Kong H, Krüger DH, Lacks S, Marinus MG, Miyahara M, Morgan RD, Murray NE, Nagaraja V, Piekarowicz A, **Pingoud A**, Raleigh E, Rao DN, Reich N, Repin VE, Selker EU, Shaw PC, Stein DC, Stoddard BL, Szybalski W, Trautner TA, Van Etten JL, Vitor JM, Wilson GG, Xu SY. (2003) A nomenclature for restriction enzymes, DNA methyltransferases, homing endonucleases and their genes. *Nucleic Acids Res.*, **31** (7), 1805–1812.
20. Wilhelm J, **Pingoud A**, Hahn M. (2003) Real-time PCR-based method for the estimation of genome sizes. *Nucleic Acids Res.*, **31** (10), e56
21. Eisenschmidt K, Lanio T, Simoncsits A, Jeltsch A, Pingoud V, Wende W, **Pingoud A**. (2005) Developing a programmed restriction endonuclease for highly specific DNA cleavage. *Nucleic Acids Res.*, **33** (22), 7039–7047.
22. Bonnet I, Biebricher A, Porté PL, Loverdo C, Bénichou O, Voituriez R, Escudé C, Wende W, **Pingoud A**, Desbiolles P. (2008) Sliding and jumping of single EcoRV restriction enzymes on non-cognate DNA. *Nucleic Acids Res.*, **36** (12), 4118–4127.
23. Midon M, Schäfer P, **Pingoud A**, Ghosh M, Moon AF, Cuneo MJ, London RE, Meiss G. (2011) Mutational and biochemical analysis of the DNA-entry nuclease EndA from Streptococcus pneumoniae. *Nucleic Acids Res.*, **39** (2), 623–634.
24. Moon AF, Midon M, Meiss G, **Pingoud A**, London RE, Pedersen LC. (2011) Structural insights into catalytic and substrate binding mechanisms of the strategic EndA nuclease from Streptococcus pneumoniae. *Nucleic Acids Res.*, **39** (7), 2943–2953.
25. Fonfara I, Curth U, **Pingoud A**, Wende W. (2012) Creating highly specific nucleases by fusion of active restriction endonucleases and catalytically inactive homing endonucleases. *Nucleic Acids Res.*, **40** (2), 847–860.
26. Schierling B, Dannemann N, Gabsalilow L, Wende W, Cathomen T, **Pingoud A**. (2012) A novel zinc-finger nuclease platform with a sequence-specific cleavage module. *Nucleic Acids Res.*, **40** (6), 2623–2638.
27. Dikić J, Menges C, Clarke S, Kokkinidis M, **Pingoud A**, Wende W, Desbiolles P. (2012) The rotation-coupled sliding of EcoRV. *Nucleic Acids Res.*, **40** (9), 4064–4070.
28. Gabsalilow L, Schierling B, Friedhoff P, **Pingoud A**, Wende W. (2013) Site- and strand-specific nicking of DNA by fusion proteins derived from MutH and I-SceI or TALE repeats. *Nucleic Acids Res.*, **41** (7), e83.
29. **Pingoud A**, Wilson GG, Wende W. (2014) Type II restriction endonucleases–a historical perspective and more. *Nucleic Acids Res.*, **42** (12), 7489–7527.

